# Association between *Pseudomonas aeruginosa* type III secretion, antibiotic resistance, and clinical outcome: a review

**DOI:** 10.1186/s13054-014-0668-9

**Published:** 2014-12-13

**Authors:** Teiji Sawa, Masaru Shimizu, Kiyoshi Moriyama, Jeanine P Wiener-Kronish

**Affiliations:** Department of Anesthesiology, Kyoto Prefectural University of Medicine, 465 Kajii-cho, Kamigyo-ku, Kyoto 602-8566 Japan; Department of Anesthesiology, School of Medicine, Kyorin University, Shinkawa 6-22, Mitaka, 181-8611 Japan; Department of Anesthesia, Critical Care and Pain Medicine, Massachusetts General Hospital, Harvard Medical University, 55 Fruit Street, GRB 44A, Boston, MA 02114 USA

## Abstract

*Pseudomonas aeruginosa* uses a complex type III secretion system to inject the toxins ExoS, ExoT, ExoU, and ExoY into the cytosol of target eukaryotic cells. This system is regulated by the exoenzyme S regulon and includes the transcriptional activator ExsA. Of the four toxins, ExoU is characterized as the major virulence factor responsible for alveolar epithelial injury in patients with *P. aeruginosa* pneumonia. Virulent strains of *P. aeruginosa* possess the *exoU* gene, whereas non-virulent strains lack this particular gene. The mechanism of virulence for the *exoU*^*+*^ genotype relies on the presence of a pathogenic gene cluster (PAPI-2) encoding *exoU* and its chaperone, *spcU*. The ExoU toxin has a patatin-like phospholipase domain in its N-terminal, exhibits phospholipase A_2_ activity, and requires a eukaryotic cell factor for activation. The C-terminal of ExoU has a ubiquitinylation mechanism of activation. This probably induces a structural change in enzymatic active sites required for phospholipase A_2_ activity. In *P. aeruginosa* clinical isolates, the *exoU*^*+*^ genotype correlates with a fluoroquinolone resistance phenotype. Additionally, poor clinical outcomes have been observed in patients with pneumonia caused by *exoU*^*+*^-fluoroquinolone-resistant isolates. Therefore, the potential exists to improve clinical outcomes in patients with *P. aeruginosa* pneumonia by identifying virulent and antimicrobial drug-resistant strains through *exoU* genotyping or ExoU protein phenotyping or both.

## Introduction

Recently, multidrug-resistant (MDR) *Pseudomonas aeruginosa* has been identified as a major cause of nosocomial infections [1,2]. *P. aeruginosa* is the most frequent Gram-negative pathogen to cause mortality of patients with ventilator-associated pneumonia (VAP) in intensive care units [3-5]. Better understanding of *P. aeruginosa* pathogenesis, and subsequent mortality, has been acquired by recent advances in knowledge regarding virulence mechanisms that lead to acute lung injury, bacteremia, and sepsis [6]. In common with other pathogenic Gram-negative bacteria, *P. aeruginosa* possesses a virulence mechanism known as the type III secretion system (TTSS). The TTSS allows the injection of toxins into the cytosol of target eukaryocytes [7,8]. The type III secretory (TTS) toxin, ExoU, has been characterized as a major virulence factor in acute lung injury [9,10]. The genomic organization of the ExoU gene, enzymatic activity of the ExoU protein, and mechanism of cell death induced by ExoU translocation have all been investigated. Among the various phenotypes of *P. aeruginosa* isolates, the ExoU-positive phenotype is a major risk factor for poor clinical outcomes. A correlation between the antimicrobial characteristics of the bacterium and an *exoU*-positive genotype has also been reported in recent clinical studies [11,12].

This review summarizes progress with respect to basic research conducted on the TTS toxin, ExoU, to date. We have covered its genomic organization and biochemistry and its ability to cause acute lung injury in people. Additionally, we will discuss the findings of recent studies on the association between ExoU and poor clinical outcome in patients.

## ExoU as a major virulence factor

**Figure 1:**
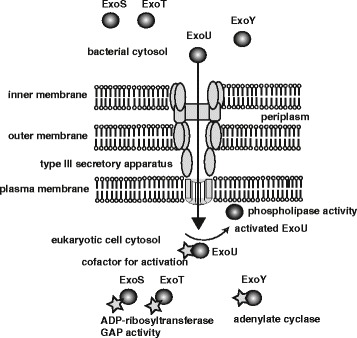
***Pseudomonas aeruginosa*** **type III secretion system.** *P. aeruginosa* injects its four type III secretory toxins ExoS, ExoT, ExoU, and ExoY directly into the cytosol of target eukaryocytes through the type III secretory apparatus. Translocated toxins are activated by specific eukaryotic cell cofactors. Following activation, ExoS shows ADP-ribosyltransferase acitivity, whereas ExoT shows ADP-ribosyltransferase and GTPase activating protein (GAP) activity. Activated ExoU has phospholipase A_2_ activity, and ExoY exhibits adenylate cyclase activity.

Isolates of *P. aeruginosa* show cytotoxicity in cultured epithelial cells and cause a high degree of acute lung injury in animal models of pneumonia [13-15]. Clinical isolates of *P. aeruginosa* display various genotypic and phenotypic variations that can affect the severity of an infection and its clinical outcome [9]. *P. aeruginosa* produces various exoproducts, among which exoenzyme S and its co-regulated proteins are candidates for cytotoxicity and acute lung injury in patients with *P. aeruginosa* pneumonia (Table 1) [16-18]. In the 1990s, based on genomic homology with its counterparts in other Gram-negative bacteria, *P. aeruginosa* exoenzyme S was identified as the effector protein that was injected into host cells via the TTSS (Figure 1) [19]. TTSSs, which are used by most pathogenic Gram-negative bacteria, including *Yersinia*, *Salmonella*, *Shigella*, *Escherichia coli*, and *P. aeruginosa*, function as molecular syringes, directly delivering toxins into the cytosol of eukaryotic cells [20]. The translocated toxins modulate eukaryotic cell signaling, a process that eventually causes disease [21,22].

**Table 1:** **Toxic protein exoproducts of** ***Pseudomonas aeruginosa***

**Exoproduct**	**Gene symbol**	**Pseudomonas genome database locus tag**	**Secretory type**	**Activity**	**Effect on host**
Alkaline protease	*aprA*	PA1249	I	Proteolysis	Blocks complement activation
Elastase (LasA and LasB)	*lasA* and *lasB*	PA1871 and PA3724	II	Elastolytic activity	Tissue destruction
Exotoxin A	*toxA*	PA1148	II	ADP-ribosyltransferase	Cytotoxin
Phospholipase C	*plcH* and *plcN*	PA0844 and PA3319	II	Phospholipase C	Heat-labile hemolysis
ExoS (exoenzyme S, 49-kDa)	*exoS*	PA3841	III	ADP-ribosyltransferase, GAP	Anti-phagocytosis
ExoT (exoenzyme S, 54-kDa	*exoT*	PA0044	III	GAP activity	Blocks wound healing
ExoU	*exoU*	-	III	Phospholipase A2	Cytotoxin, anti-phagocytosis
ExoY	*exoY*	PA2191	III	Adenylate cyclase	Edema formation

GAP, GTPase activating protein activity.

**Figure 2:**
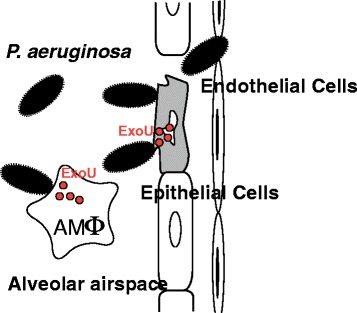
**Alveolar epithelial injury caused by the ExoU cytotoxin of** ***Pseudomonas aeruginosa*** **.** Cytotoxic *P. aeruginosa* in the respiratory airspace injects the ExoU cytotoxin into alveolar epithelial cells, destroys the integrity of lung epithelium, and disseminates into the systemic circulation, causing bacteremia and sepsis.

PA103 lacks the exoenzyme S gene (*exoS*) encoding the 49-kDa form of the toxin but possesses the exoenzyme T gene (*exoT*), which encodes the 53-kDa form. An isogenic mutant missing *exoT* was found to be cytotoxic to cultured epithelial cells and caused acute lung injury; therefore, it was concluded that neither ExoT nor ExoS was a major virulence factor for lung injury [18]. PA103 was found to secrete a unique unknown 74-kDa protein, the production of which was decreased when a transposon mutation in *exsA* was present. The gene encoding this protein was cloned, and a mutant lacking this protein was created in PA103. The isogenic mutant lacking the 74-kDa protein failed to cause acute lung injury in animal models [9]. This protein, regulated by ExsA, a transcriptional activator of *P. aeruginosa* TTSS, was designated ExoU [9,23]. Along with other TTS toxins, such as ExoS and ExoT, ExoU is secreted through the TTSS and injected directly into the cytosol of targeted eukaryocytes. Clinical isolates with cytotoxic phenotypes *in vitro* were found to possess *exoU*, whereas non-cytotoxic isolates lacked *exoU* [24]. Additionally, cytotoxic clinical isolates secreting ExoU caused severe and acute epithelial injury in animal models of *P. aeruginosa* pneumonia (Figure 2) [24]. It was postulated that the ability of *P. aeruginosa* to cause acute lung epithelial injury and sepsis is strongly linked to TTS secretion of ExoU [10].

## Genomic organization of ExoU

**Figure 3:**
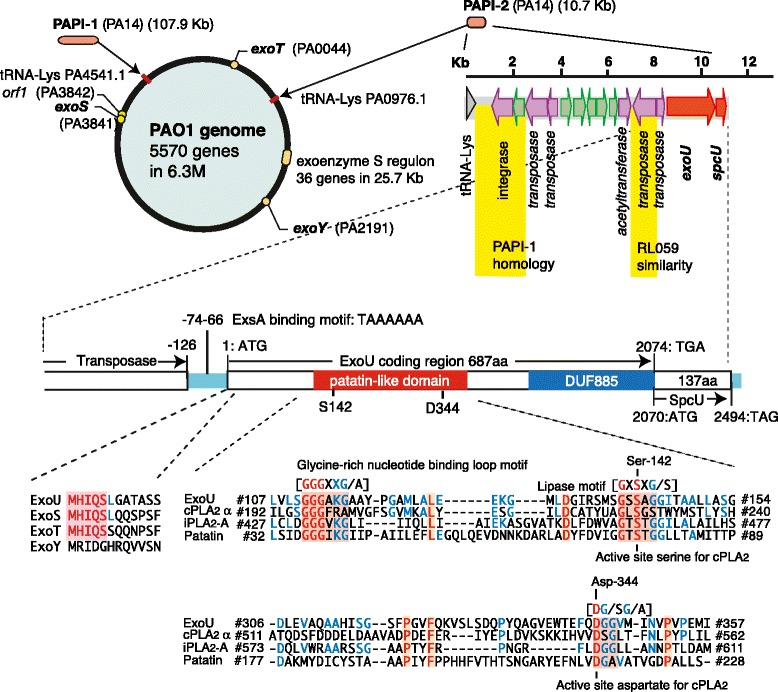
**Genomic organization of PAPI-2 and** ***exoU*** **.** Genomic organization of the *Pseudomonas aeruginosa* strain, PAO1, is shown (upper left). PAO1 contains 5,570 open reading frames (ORFs) in its 6.3-Mb genome. The type III secretion regulatory region (25.5 kb), known as the exoenzyme S regulon, contains 36 genes in the five operons. Genes encoding the type III secretory toxins *exoS*, *exoT*, and *exoY*, but not *exoU*, are scattered throughout the genome. *exoU* is located in an insertional pathogenic gene cluster known as PAPI-2 [26,30]. PAPI-2 has 14 ORFs, including *exoU* and *spcU*, in a 10.7-Kb region (upper right). *exoU* has an ORF of 2,074 base pairs and a promoter region with an ExsA binding motif (TAAAAAA) at -74 (lower middle). The primary sequence of ExoU is 682 amino acids and contains an N-terminal secretory leader sequence, which shares similarity with ExoS and ExoT, and a patatin-like phospholipase domain with a catalytic dyad (Ser142 and Asp344), and a C-terminal DUF885 domain associated with a proposed ubiquitin binding domain. The patatin-like domain of ExoU has a structure similar to cytosolic phospholipase A_2_ (cPLA_2_), calcium-independent PLA_2_ (iPLA_2_), and patatin from plants. Red letters with highlighted backgrounds indicate a perfect match. Blue letters indicate a partial match for amino acid sequences among four PLA_2_ proteins. PAPI, *Pseudomonas aeruginosa* pathogenicity island.

*P. aeruginosa* strain PAO1 was the first strain whose genome was completely sequenced in 2001 by the *Pseudomonas* Genome Project. A pathogenic gene cluster, the exoenzyme S regulon, encodes genes underlying the regulation, secretion, and translocation of the TTSS. In the exoenzyme S regulon, five operons (*exsD–pscL*, *exsCBA*, *pscG–popD*, *popN–pcrR*, and *pscN–pscU*) encode TTSS and translocation machinery. The *exsCBA* operon encodes the transcriptional activator protein ExsA, which regulates expression of exoenzyme S and co-regulated proteins. The PAO1 strain lacks *exoU*, whereas approximately 20% of clinical isolates possess *exoU* (Figure 3) [25]. The *exoU* gene was initially cloned from the PA103 strain, along with its cognate chaperone gene *spcU* [9]. The genomic organization of the ExoU-secreting clinical isolate PA14 was analyzed, and two insertional genomic islands, termed pathogenicity islands PAPI-1 and PAPI-2 (*Pseudomonas aeruginosa* pathogenicity island), were discovered (Figure 3) [26]. The 10.7-kb PAPI-2 region, which is probably derived via horizontal gene transfer, lies within the tRNA-Lys (PA0976.1) region (Figure 3); it encodes 14 open reading frames, including *exoU*, *spcU*, four transposases, one integrase, one acetyltransferase, and six hypothetical proteins. The *exoU* gene itself is 2,074 base pairs and encodes the 682 amino acid protein, ExoU [27] (Figure 3). Four nucleotides at the 3′ end, including the stop codon in *exoU*, overlap the start codon of the 324-base pair *spcU* gene, which encodes SpcU (137 amino acids). The promoter region of *exoU* has a binding motif (TXAAAAXA) for the transcriptional activator, ExsA [28,29].

## Enzymatic action of ExoU

**Figure 4:**
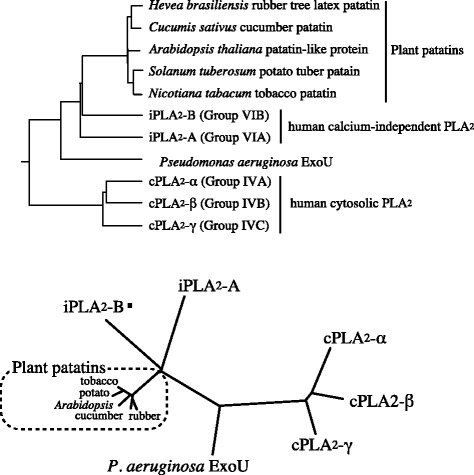
**Phylogenetic analysis of lipase domains in patatin-like phospholipases.** The conserved domain of ExoU contains a patatin-like phospholipase motif. Patatin, a storage protein in potato tubers, cucumbers, and rubber latex, exhibits lipase activity and shares a catalytic dyad structure with mammalian phospholipase A_2_ (PLA_2_). The catalytic domains of ExoU align with those of patatin, human calcium-independent PLA_2_ (iPLA_2_) and cytosolic PLA_2_ (cPLA_2_). Phylogenetic analysis was conducted by using ClustalW [39].

**Figure 5:**
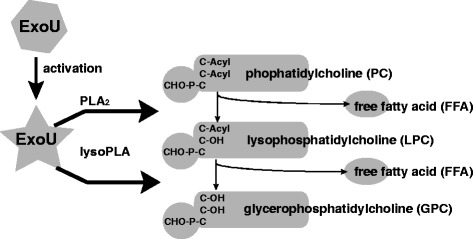
**Phospholipase A**_**2**_ **and lysopholipase A activity of activated ExoU.** ExoU that is activated by eukaryotic cell cofactors after translocation into the eukaryotic cell cytosol demonstrates hydrolytic activity toward phospholipids via its phospholipase A_2_ (PLA_2_) and lysophospholipase (lysoPLA) activities.

The N-terminal of ExoU starts at the secretory leader (MHIQS), the sequence of which is the same as the starter sequence for ExoS and ExoT. When ExoU was identified as a major virulence factor causing acute lung injury in 1997, little was known about its enzymatic mechanisms that were responsible for acute cell death. Analysis of the conserved domain of ExoU revealed a patatin-like domain, containing a glycine-rich nucleotide binding loop motif and a lipase motif with catalytically active serine and aspartate within its N-terminal primary sequence [31]. Patatin, a storage protein in potatoes, exhibits lipase activity and shares a catalytic dyad structure with mammalian phospholipase A_2_ (PLA_2_) (Figures 3 and 4) [32-35]. The catalytic domains of ExoU align with those of patatin, human calcium-independent PLA_2_ (iPLA_2_) and cytosolic PLA_2_ (cPLA_2_) [36]. The predicted active sites for ExoU PLA_2_ activity are serine 142 (S142) and aspartate 344 (D344). Site-directional mutagenesis of the predicted catalytic residues (ExoUS142A or ExoUD344A) eliminated the cytotoxicity of PA103 [36,37]. Inhibitors of iPLA_2_ and cPLA_2_, including bromoenol lactone (BEL), methyl arachidonyl fluorophosphate (MAFP), and arachidonyl trifluoromethyl ketone (AACOCF_3_), reduced the cytotoxicity of PA103 *in vitro*. In the presence of a eukaryotic cell extract, recombinant ExoU displayed PLA_2_ and lysophospholipase (lysoPLA) activities (Figure 5); these activities were inhibited by cPLA_2_ or iPLA_2_ inhibitors [31,38]. The site-directional PA103 mutants lacking PLA_2_ activity were tested by using an animal model of pneumonia. In PA103, either of the ExoUS142A or ExoUD344A mutations abolished virulence associated with acute lung injury and death. It was concluded that acute lung injury from cytotoxic *P. aeruginosa* is caused by the cytotoxic activity of the patatin-like phospholipase domain of ExoU.

ExoU displays serine acylhydrolase activity via a Ser/Asp catalytic dyad and can be classified as a group IV PLA_2_ member. A major characteristic of serine acylhydrolases, such as PLA_2_, PLA_1_, and lysoPLA, is their ability to perform multiple lipase reactions [40]. Recently, more patatin-like PLA_2_ proteins have been detected in various bacterial species [41]. It seems likely that bacteria use PLA_2_ as a defense mechanism against predatory eukaryocytes such as phagocytes and environmental amoeba. Its presence allows them to attack a target cell to obtain nutrition, thereby increasing their population [40]. ExoU can kill eukaryotic predators, such as the amoeba *Acanthamoeba castellanii* [42,43]. Intracellular expression of ExoU is cytotoxic to yeast, suggesting that fungi could be one of its potential targets [44]. In humans, *P. aeruginosa* targets phagocytic cells in the lungs and injects them with ExoU [45-47]. In an animal model of pneumonia, ExoU is produced during the early phase of infection; delaying *exoU* expression by as little as 3 hours enhanced bacterial clearance and survival of infected mice [48]. ExoU-mediated impairment of phagocytes probably allows *P. aeruginosa* to persist within the lungs, causing localized immunosuppression and facilitating superinfection with less pathogenic bacteria. This would explain not only why ExoU-secreting *P. aeruginosa* is associated with more severe pulmonary infections but also the tendency of hospital-acquired pneumonia to be polymicrobial [47].

## ExoU cytotoxicity and its various effects

Non-cytotoxic *P. aeruginosa* strains transformed with pUCP19*exoUspcU*, a plasmid that carries *exoU* and *spcU*, became cytotoxic to cultured epithelial cells *in vitro* and lethal in a mouse model of pneumonia [49]. Isogenic mutants, generated to secrete ExoU, ExoS, or ExoT, were evaluated for their relative contributions to pathogenesis in a mouse model of acute pneumonia [50]. In this study, measurements of mortality, bacterial persistence in the lungs, and dissemination of the bacteria indicated that ExoU secretion had the greatest impact on virulence but that secretion of ExoS had a moderate effect and ExoT a relatively minor effect.

ExoU translocation induces cell death by destroying cell membranes via PLA_2_ activity. ExoU might also contribute to the induction of an eicosanoid-mediated inflammatory response in host organisms, as airway epithelial cells exposed to *P. aeruginosa* overproduce prostaglandin E_2_ in an ExoU-dependent manner [51,52]. A deleterious effect on phospholipid metabolism, in concert with caspase activation, was also reported to occur in an ExoU-dependent manner [53]. Another study reported that arachidonic acid-induced oxidative stress might cause cell damage during the course of an ExoU-producing *P. aeruginosa* infection. This is because endothelial cell death in cytotoxic PA103 infections was significantly attenuated by alpha-tocopherol [54]. ExoU could also contribute to the pathogenesis of lung injury as it induces a tissue factor-dependent procoagulant activity in airway epithelial cells [55], vascular hyperpermeability, platelet activation, and thrombus formation during *P. aeruginosa* pneumonia and sepsis [56].

## Activation mechanism of ExoU

TTS toxins use a unique mechanism for activating their enzymatic activities. These toxins are initially produced in the bacterial cytosol as inactive forms and, immediately after being injected into the cytosol of a target eukaryotic cell by the bacterial secretion apparatus, are activated by specific eukaryotic cell cofactors. As an example, ExoS ADP-ribosyltransferase activity is activated by the eukaryotic protein factor FAS (factor activating exoenzyme S), which is a member of the 14-3-3 protein family [57,58]. In contrast, *P. aeruginosa* adenylate cyclase ExoY requires an unknown eukaryotic cell factor for its activation [59]. The PLA_2_ activity of ExoU cleaves plasma membrane phospholipids and causes the rapid lysis of targeted eukaryotic cells. Similar to ExoS and ExoY, ExoU requires eukaryotic cell cofactors for its activation, whereas *in vitro* PLA_2_ assays with recombinant ExoU require the addition of eukaryotic cell lysates. The patatin-like PLA_2_ domain is located at the N-terminal region of ExoU; the C-terminal region, which includes a sequence corresponding to a conserved DUF885 domain, was reported to be important for the activation process and membrane localization of the protein [60-62]. In 2006, Sato and colleagues [63] reported that Cu^2+^, Zn^2+^-superoxide dismutase (SOD1) was a cofactor that activated the PLA_2_ activity of ExoU. By this time, however, it had also been reported that ExoU localizes to the plasma membrane, where it undergoes modification in the cell by the addition of two ubiquitin molecules at lysine 178; five C-terminal residues (679 to 683) control membrane localization and ubiquitination [64]. Site-directed spin-labeling electron paramagnetic resonance spectroscopy revealed that the addition of SOD1 induced conformational changes in ExoU [65]. PLA_2_ activity of ExoU was demonstrated by using ubiquitinated yeast SOD1 and other ubiquitinated mammalian proteins [66]. Therefore, it seems that ubiquitinated SOD1 works as a ubiquitin donor and that ubiquitination of the ExoU C-terminal activates the PLA_2_ activity of ExoU.

**Figure 6:**
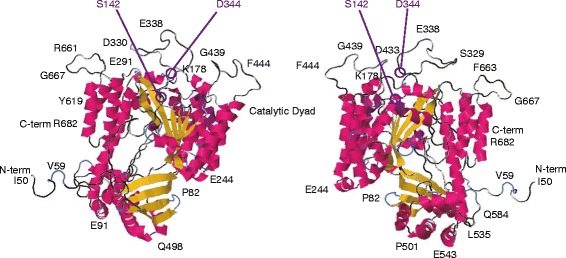
**Three-dimensional structure of** ***Pseudomonas aeruginosa*** **ExoU.** Left: position at 0; right: position horizontally rotated −180°. The phospholipase A_2_ catalytic dyad comprises S142 and D344. Loop structures such as the K178 area, S329-D344, G439-F444, and Y619-R682 seem to affect the structure of the catalytic dyad. The proposed ubiquitin-binding domain at the C-terminal of ExoU might comprise a pocket structure from these loops. Images of three-dimensional structures were generated by using the protein structure prediction server RaptorX [72].

The three-dimensional crystallographic structure of ExoU combined with its cognate chaperone SpcU was recently elucidated by two research groups [67,68] (Figure 6). In one of these studies, the C-terminal membrane-binding domain of ExoU displayed specificity for phosphatidylinositol 4,5-bisphosphate (PI_4,5_P_2_); ubiquitination of ExoU resulted in its co-localization with endosomal markers [67]. The ubiquitin-binding domain was mapped to a C-terminal four-helix bundle in ExoU [69], with PI_4,5_P_2_ synergistically enhancing the PLA_2_ activity of ExoU via a ubiquitin-related mechanism [70] (Figure 6). The *Rickettsia prowazekii* RP534 protein, a homologue of ExoU, possesses PLA_2_ and lysoPLA activities and PLA_1_ activity in the absence of any eukaryotic cofactors [71]. A structural comparison between ExoU and RP534 protein would help clarify the ubiquitin-associated mechanism of ExoU activation. Research into the mechanisms of ExoU activation has provided new insights into how bacteria manipulate eukaryotic cell signaling to facilitate their growth and pathogenesis.

## Clinical epidemiology of *Pseudomonas aeruginosa* type III secretory-associated genotypes

Early studies on *P. aeruginosa* TTSS revealed an association between a cytotoxic or invasive phenotype and genotype of a strain. The invasive PAO1 strain and the cytotoxic PA103 strain harbor the *exoS*^*+*^*exoT*^*+*^*exoU*^*−*^ and *exoS*^*−*^*exoT*^*+*^*exoU*^*+*^ genotypes, respectively [9,18]. This genetic variation in TTS toxin genes implies the presence of similar genotypic and phenotypic variations among clinical and environmental isolates [73]. Consequently, isolates from the respiratory tract or blood cultures of 108 patients were analyzed, and the relative risk of mortality was reported to be sixfold greater when expression of ExoS, ExoT, ExoU, or PcrV occurred (Table 2). The prevalence of the TTS-positive phenotype was significantly higher in acutely infected patients than in chronically infected cystic fibrosis (CF) patients [24]. When Schulert and colleagues [74] analyzed the virulence profiles of 35 *P. aeruginosa* isolates from patients with hospital-acquired pneumonia by using a cytolytic cell-death assay, an apoptosis assay, and a mouse model of pneumonia, they found that increased virulence was associated with the secretion of ExoU but not ExoS or ExoY secretion. These studies suggest that *P. aeruginosa* TTSS is present in nearly all clinical and environmental isolates. ExoU secretion could be used as a marker for highly virulent strains and could have some association with poor clinical outcome. It appears that isolates from acutely infected patients are genotypically different from those from chronically infected CF patients [73]. Other researchers have reported the presence of different *P. aeruginosa* genotypes in isolates from CF patients. The *exoS*^*+*^*exoU*^−^ genotype is associated with chronic infection in CF patients, whereas the *exoS*^*−*^*exoU*^+^ genotype is associated with bacterial strains isolated from blood [75-79].

**Table 2:** **Associations between the** ***Pseudomonas aeruginosa*** **type III secretion system and clinical outcomes**

**Reference**	**Year**	**Country**	**Target population**	**Clinical association**
Roy-Burman *et al*. [24]	2001	USA	108 isolates from respiratory tract or blood	TTSS-positive phenotype was a predictor of poor clinical outcome.
Hauser *et al*. [80]	2002	USA	35 patients with VAP	In VAP, type III secretory isolates were associated with worse clinical outcomes.
Schulert *et al*. [74]	2003	USA	35 isolates from patients with hospital-acquired pneumonia	ExoU is a marker for highly virulent strains.
Wareham and Curtis [75]	2007	UK	TTSS genotypes and phenotypes of 163 clinical isolates	The *exoS* ^−^/*exoU* ^+^ genotype was associated with strains isolated from blood.
Garey *et al*. [81]	2008	USA	Hospitalized patients with bacteremia	Mortality did not differ among patients infected with *exoS* or *exoU* isolates.
Wong-Beringer *et al*. [12]	2008	USA	45 isolates susceptible to fluoroquinolones	*exoU* ^+^ strains exhibited increased cytotoxicity compared with ExoS-secreting strains.
Bradbury *et al*. [76]	2010	Australia	184 clinical, nosocomial, and environmental isolates	Isolates collected from the environment of intensive therapy units were more likely to possess *exoU*.
Agnello and Wong-Beringer [82]	2012	USA	270 respiratory isolates	Strains with fluoroquinolone resistance correlate with TTSS effector genotype and the more virulent *exoU* ^+^ subpopulation.
El-Solh *et al*. [83]	2012	USA	85 cases of bloodstream infection	Expression of TTSS toxins in isolates from bacteremic patients confers poor clinical outcomes.
Jabalameli *et al*. [84]	2012	Iran	96 isolates collected from wound infections of burn patients	*exoU* gene is disseminated among isolates from burn patients.
Sullivan *et al*. [11]	2014	USA	218 adult patients with positive respiratory cultures	Fluoroquinolone-resistant phenotype in *exoU* strains contributes to pneumonia.

TTSS, type III secretion system; VAP, ventilator-associated pneumonia.

## Clinical epidemiology associated with ExoU and antibiotic resistance

Another important topic in *P. aeruginosa* biology that has recently emerged is the association of antibiotic resistance with TTSS virulence genotypes (Table 2). Mitov and colleagues [85] analyzed the antimicrobial resistance profiles and genotypes of 202 isolates from CF patients (n = 42) and non-CF in-patients (n = 160). The authors found that the prevalences of *exoS* and *exoU* were 62.4 and 30.2%, respectively, and that *exoU* was more prevalent among MDR than in non-MDR strains (40.2% versus 17.7%). Garey and colleagues [81] reported that 97.5% of bloodstream isolates harbored *exoS* or *exoU* genes and that *exoS* was the most prevalent (70.5%; n = 86). The prevalence of *exoU* was 25.4% (n = 31), and these isolates were significantly more likely to be resistant to multiple antibiotics, including cephems, carbapenems, fluoroquinolones, and gentamicin. Consistent with this, an analysis of 45 clinical isolates found that *exoU*^*+*^ isolates were more likely to be fluoroquinolone-resistant than *exoS*^*+*^ isolates (92% versus 61%, *P* <0.05). These isolates possessed a mutation in the *gyrA* gene and exhibited an efflux pump overexpression phenotype [12]. Agnello and Wong-Beringer [82] examined the relationship between the TTSS effector genotype and fluoroquinolone resistance mechanisms in 270 respiratory isolates and found that a higher proportion of *exoU*^*+*^ strains was fluoroquinolone-resistant compared with *exoS*^*+*^ strains (63% versus 49%) despite their lower prevalence (38% *exoU*^*+*^ versus 56% *exoS*^*+*^) [82]. Of epidemiological importance, Tran and colleagues [86] showed that 20 isolates (eight unique pulsed-field gel electrophoresis clusters) recovered from imported frozen raw shrimp sold in the US harbored TTS toxin genes and were resistant to quinolone with mutations in *gyrA*. These findings indicate co-evolution of resistance and virulence traits favoring a more virulent genotype in a quinolone-rich clinical environment [80].

There have been several studies in which associations between TTSS-associated virulence and poor clinical outcome for *P. aeruginosa*-infected patients have been observed. An analysis of TTS genotypes and phenotypes of isolates cultured from 35 mechanically ventilated patients with bronchoscopically confirmed *P. aeruginosa*-VAP showed a correlation between TTS phenotype, especially the ExoU phenotype, and severity of pneumonia [80]. More recently, El-Solh and colleagues [83] performed a retrospective analysis of 85 cases of *P. aeruginosa* bacteremia. Bacteremic patients with TTSS-positive isolates developed septic shock with high probability of death more frequently than patients with TTSS-negative isolates. The authors found that none of the TTSS-positive patients who survived the first 30 days of infection had a *P. aeruginosa* isolate that exhibited the ExoU phenotype; a higher frequency of antibiotic resistance was detected in TTSS-positive isolates. Jabalameli and colleagues [84] analyzed TTSS genotypes and antimicrobial resistance in 96 isolates collected from wound infections of burn patients. More than 90% of the isolates were MDR, and 64.5% of them carried *exoU* whereas 29% carried *exoS*. Their findings suggest that these genes, particularly *exoU*, are commonly disseminated among *P. aeruginosa* strains isolated from burn patients. Sullivan and colleagues [11] recently reported their analysis of antimicrobial resistance and TTSS virulence in *P. aeruginosa* isolates from hospitalized adult patients with respiratory syndromes. The authors studied 218 consecutive adult patients whose respiratory cultures were positive for *P. aeruginosa*, and reported that fluoroquinolone-resistant and MDR strains were more likely to cause pneumonia than bronchitis or colonization. The combination of fluoroquinolone resistance and the gene encoding the TTSS ExoU effector in *P. aeruginosa* was the strongest predictor of pneumonia development. Further investigations suggest that the fluoroquinolone-resistant phenotype and the *exoU*^+^ genotype of *P. aeruginosa* might cause poor clinical outcomes in patients with *P. aeruginosa* pneumonia [87]. Although there is no clear genetic explanation and a less than convincing association between ExoU-associated virulence and antibiotic resistance, there is no doubt that bacterial strains possessing both virulent and MDR characteristics are more dangerous, especially for immunocompromised patients. Therefore, improved genotyping or phenotyping methods (or both) for analyzing TTS toxins of clinical isolates will enhance our understanding of this area.

## Potential therapeutic strategies against ExoU-derived cytotoxicity

Several prophylactic or therapeutic experimental strategies against the cytotoxic effects of TTS ExoU have been reported over the last decade. The *P. aeruginosa* V-antigen PcrV, a homolog of the *Yersinia* V-antigen LcrV, contributes to TTS toxin translocation [88]. In prophylactic strategies, active immunization against PcrV ensures the survival of challenged mice and decreases lung inflammation and injury [89]. DNA vaccination with pIRES-toxAm-pcrV has been proposed as a potential immunotherapy [90]. In passive immunization, the rabbit polyclonal anti-PcrV antibody and murine monoclonal anti-PcrV antibody mAb166 inhibit TTS toxin translocation [91-95]. For clinical use, the mAb166 was humanized [96], and the IgG antigen-binding (Fab′) fragment, KB001, is currently in use in phase II clinical trials for treating VAP in France and chronic pneumonia in CF patients in the US [97,98].

*In vitro* experiments have shown that specific inhibitors against iPLA2, such as BEL, AACOCF3, and MAFP, decrease the cytotoxicity of ExoU. Several researchers have reported that small molecules, such as pseudolipasin A and arylsulfonamides, specifically inhibit the phospholipase activity of ExoU [99,100]. More details regarding the activation mechanisms of ExoU have been recently reported; however, there is more potential in using small chemicals for the prevention of acute lung injury induced by *P. aeruginosa*.

## Conclusions

*P. aeruginosa* ExoU, a toxin injected into the cytosol of target eukaryotic cells such as phagocytes and epithelial cells, is a major virulence factor in the cause of alveolar lung injury in patients with *P. aeruginosa* pneumonia. Virulent strains of *P. aeruginosa* possess the PAPI-2 pathogenic gene cluster region, which includes *exoU*. The PLA_2_ activity exhibited by ExoU requires a ubiquitination-associated activation mechanism to operate in a eukaryotic cell factor-dependent manner. A combination of the *exoU*^*+*^ genotype and fluoroquinolone-resistant phenotype in isolates was shown to correlate with poor clinical outcome. Cytotoxic and antimicrobial-resistant *P. aeruginosa* is a serious concern, especially for immunocompromised patients. Therefore, rapid diagnostic determination of isolate genotype and phenotype is important. Surveillance to determine the prevalence of cytotoxic and antibiotic-resistant isolates is needed if we are to reduce the risk of lethal *P. aeruginosa* outbreaks. Opportunities exist for improving the clinical outcome of patients infected with *P. aeruginosa* by identifying virulent and antimicrobial-resistant isolates that cause acute lung injury, sepsis, and mortality. Exploration of *P. aeruginosa* virulence apparatuses as potential antimicrobial targets is vital if we are to avoid the spread of dangerous super-resistant *P. aeruginosa* strains.

## Abbreviations

AACOCF_3_: Arachidonyl trifluoromethyl ketone
BEL: Bromoenol lactone
CF: Cystic fibrosis
cPLA_2_: Cytosolic phospholipase A_2_
iPLA_2_: Calcium-independent phospholipase A_2_
lysoPLA: Lysophospholipase
MAFP: Methyl arachidonyl fluorophosphate
MDR: Multidrug-resistant
PAPI: *Pseudomonas aeruginosa* pathogenicity island
PI_4,5_P_2_: Phosphatidylinositol 4,5-bisphosphate
PLA_2_: Phospholipase A_2_
SOD1: Superoxide dismutase 1
TTS: Type III secretory
TTSS: Type III secretion system
VAP: Ventilator-associated pneumonia
